# Overcrowding and Hazardous Dwelling Condition Characteristics: A Systematic Search and Scoping Review of Relevance for Health

**DOI:** 10.3390/ijerph192315542

**Published:** 2022-11-23

**Authors:** Johnny C. Lorentzen, Gunnar Johanson, Folke Björk, Sofia Stensson

**Affiliations:** 1 Institute of Environmental Medicine, Integrative Toxicology, Karolinska Institutet, SE-171 77 Stockholm, Sweden; 2 Center for Occupational and Environmental Medicine, Region Stockholm, SE-113 65 Stockholm, Sweden; 3 KTH Royal Institute of Technology, SE-100 44 Stockholm, Sweden; 4 RISE Research Institutes of Sweden, SE-501 15 Borås, Sweden

**Keywords:** crowding, overcrowding, mold, biocides, exposure, confounding, pesticides

## Abstract

Crowding in dwellings is an important public health issue. We hypothesize that overcrowding may cause indirect health effects by adversely affecting the dwelling itself, for example, by increasing dampness leading to mold. We therefore performed a systematic search and a scoping review on overcrowding leading to dwelling condition characteristics of relevance for health. A literature search was performed using the PubMed and Scopus databases up to 5 March 2021. The search yielded 100 records with relevant information. We found that overcrowding is defined in numerous ways and often address “socially deprived” populations. Six studies report associations of overcrowding with at least one dwelling condition characteristic, namely lead, cadmium, microorganism distribution, dust mite and cockroach allergens in dust, cockroach infestation, peeling paint, and mold. One of the studies reports associations between several characteristics, e.g., association of mold with cleanliness and rodent infestation, and points out the common use of pesticides. Additional characteristics were extracted from the remaining 94 records, without data on statistical associations with overcrowding. Our review suggests that multiple potentially hazardous dwelling condition characteristics often coincide in overcrowded dwellings. The epidemiological attribution of health effects to any characteristic is therefore difficult. Causal relationships are even more difficult to establish, as overcrowding is also associated with a range of social and other circumstances that may affect health. The complexity should be considered by scientists and practitioners dealing with overcrowding in dwellings.

## 1. Introduction

Article 25 of The United Nations Universal Declaration of Human Rights states that “Everyone has the right to a standard of living adequate for the health and well-being of himself and of his family, including …..housing” [1]. In line with this, the “Housing and Health Guidelines” by the World Health Organization (WHO) highlights housing as an important public health issue, based on systematic literature reviews up to 2018 [2]. One chapter in the guidelines deals with household crowding and points to key health outcomes of interest for further studies, i.e., tuberculosis and other infectious diseases, gastroenteritis and diarrheal diseases, sleep quality, intimate partner violence, and mental health. Another chapter, which deals with low temperatures and insulation, informs that water vapor produced by human metabolism and household activities may, in the absence of adequate ventilation, lead to increased humidity and growth of mold. Consequently, too many residents in a dwelling according to codes and norms, i.e., overcrowding, may increase indoor humidity and lead to potentially hazardous mold.

**Figure 1:**
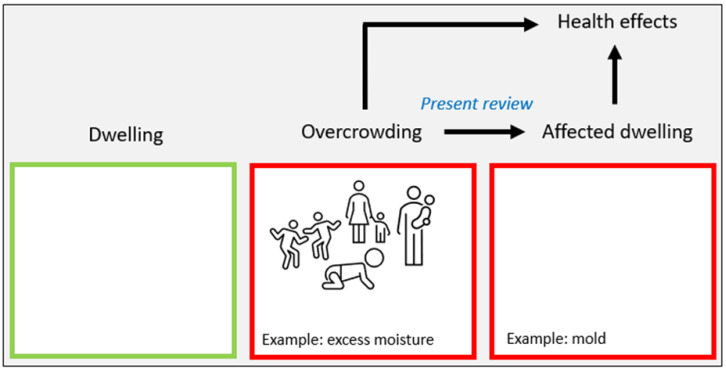
Outline of our underlying hypothesis in the present review. Overcrowding may affect dwellings, leading to dwelling condition characteristics of relevance for health, as exemplified by excess humidity (dampness/moisture) leading to mold. Thus, we did not review associations between overcrowding and health.

We hypothesize that overcrowding, in addition to mold, may lead to several biological, chemical, and physical exposures of relevance for health (Figure 1). The identification and awareness of such hazardous environmental exposures is important for taking rational measures to prevent or reduce their adverse impact on health. The aim in this review was to extract and synthesize information from the literature on overcrowding leading to hazardous dwelling condition characteristics.

## 2. Materials and Methods

### 2.1. Systematic Review

Our review followed the Preferred Reporting Items for Systematic Reviews and Meta-Analyses (PRISMA) guidelines [3] and was registered (CRD42020126810) in the PROSPERO international database of prospectively registered systematic reviews. The registered review question, posed in relation to health, was *“which characteristics of dwelling conditions are described in connection to the dwellings being overcrowded?”*

### 2.2. Identification and Screening of Records

The PubMed and Scopus databases were searched (up to 5 March 2021) for records on overcrowding (or similar descriptors), in dwellings (or similar descriptors), and in current member countries of The Organization for Economic Cooperation and Development (OECD) (see Appendix A: Search strategy). The title and abstract had to be in English. Identified records were imported in Rayyan, an online tool for systematic review (https://www.rayyan.ai, accessed 21 November 2022) and duplicates were removed. Using Rayyan, two investigators (authors J.C.L. and F.B.) independently screened the titles and abstracts of all records (other text if abstract not available) and excluded those that did not mention any dwelling condition characteristic. The characteristics had to be something specific (e.g., mold) or non-specific (e.g., “unclean”) that will remain in the dwelling after the evacuation of residents and their belongings, thus including effects on the dwelling construction and interiors but excluding, e.g., moisture/dampness in materials and air, and airborne contaminants. Cases of disagreement were solved by discussion, ending in consensus.

### 2.3. Assessing Records for Eligibility, Including Records Identified during Assessment

Two investigators (authors J.C.L., F.B., G.J., and S.S., in different combinations) independently assessed the remaining records for eligibility. Records were excluded if the full text lacked relevant information. During assessment, the reference lists and texts were screened for additional records, and those identified by hand searching were also assessed for eligibility. Cases of disagreement were solved by discussion with all four authors, ending in consensus.

### 2.4. Data Extraction and Synthesis of Eligible Studies

Any content deemed relevant was extracted and summarized. The records were divided in two categories according to the level of information regarding overcrowding and dwelling condition characteristic(s). Thus, records presenting quantitative information on both variables were assigned as category A, while those lacking quantitative information on one or both variables were assigned as category B. Categorizations and data extractions were performed independently by at least two authors and cases of disagreement were solved by discussion with all four authors, ending in consensus. Category A studies were scrutinized from four aspects: (1) descriptions and definitions of overcrowding, (2) description of dwelling condition characteristics, (3) numerical measures of the two variables, and (4) quantitative associations between the two variables. Each study was condensed to a short description of the four aspects. From records in Category B, any content deemed relevant was extracted. Finally, an overall synthesis was made.

## 3. Results

### 3.1. Identification, Screening, and Assessment of Records for Eligibility

**Figure 2:**
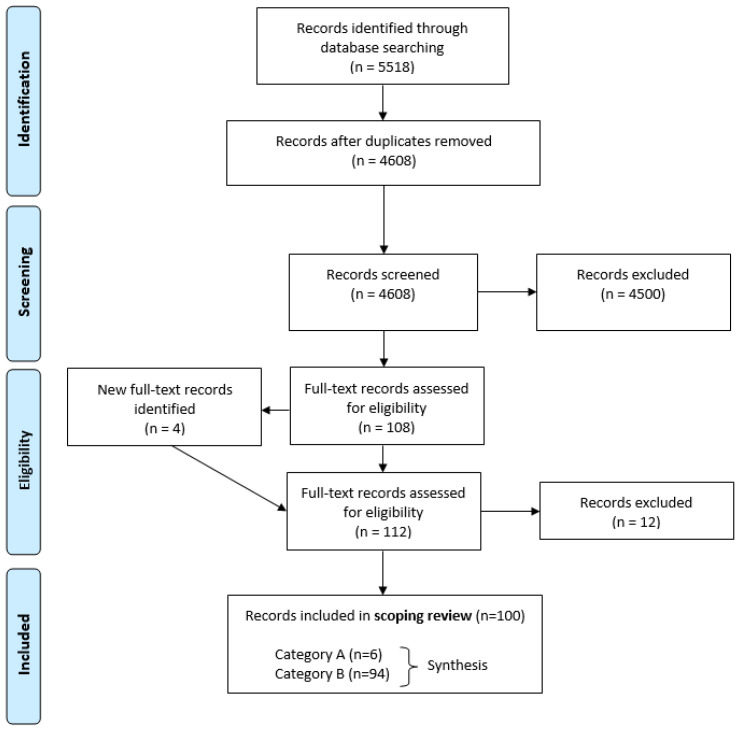
Literature search, screening results, and analysis displayed as a PRISMA flow diagram. Figure design adapted from Moher et al. 2009 [3]. Note: PRISMA, Preferred Reporting Items for Systematic Reviews and Meta-Analyses (http://www.prisma-statement.org).

A total of 5518 of records were identified by searches in PubMed (*n* = 2313) and Scopus (*n* = 3205); this was reduced to 4608 unique records after the removal of duplicates (Figure 2). Most of the records were excluded (*n* = 4500) during screening of abstracts and titles (or other available text) for various reasons. For example, some records were excluded because they addressed irrelevant topics, such as crowdfunding, crowdsourcing, crowding of animals, or crowding of people outside dwellings. Nonetheless, most of the excluded records dealt with the intended meaning of overcrowding in dwellings; however, the title/abstract did not mention any dwelling condition characteristic. Following this first screening, 108 records remained; these were retrieved in full-text format and assessed for eligibility. During this procedure, four additional records were identified, retrieved as full-texts, and assessed, giving a total of 112 records. Finally, 12 records were excluded as the content selected for in the title and abstract did not correspond to the content in the full text. Thus, our record identification, screening, and assessment resulted in the inclusion of 100 records [4,5,6,7,8,9,10,11,12,13,14,15,16,17,18,19,20,21,22,23,24,25,26,27,28,29,30,31,32,33,34,35,36,37,38,39,40,41,42,43,44,45,46,47,48,49,50,51,52,53,54,55,56,57,58,59,60,61,62,63,64,65,66,67,68,69,70,71,72,73,74,75,76,77,78,79,80,81,82,83,84,85,86,87,88,89,90,91,92,93,94,95,96,97,98,99,100,101,102,103] for data extraction and synthesis (Figure 2).

### 3.2. Description of Eligible Records, Data Extraction, and Categorization

The 100 eligible records (Appendix B: Table A1,) covered different aspects of public health but are otherwise highly heterogenous, e.g., in the scope, methods used, and types of text, e.g., research articles, reviews [6,12,17,33,41,43,48,52,57,60,62,99,101], overviews, syntheses [30,63], position statement [87], discussion paper [45], perspectives [37,77], book chapter [4], proceedings [76], conference paper [26], and reports/studies from authorities [27,56]. The papers are published between 2020 and 1976, some being historical accounts. For example, one paper describes Dublin as the unhealthiest major city in Britain and Ireland around 1850 [4], and another pictures filth and disorder in Toronto slums around 1910 [18]. We note that overcrowding is defined in numerous ways (Appendix B: Table A2) and that “socially deprived” residents are often in focus. At least 36 records address ethnic dimensions, and many of these deal with migrants, immigrants, or indigenous populations (Appendix B: Table A1). Four full texts were non-English, namely Turkish [11], Italian [22], Hungarian [46], or Spanish [103]. Taken together, the 100 records describe dwellings condition characteristics in more than 20 OECD countries (Appendix B: Table A1). Their content is reflected by the abstracts, which often mention, in different wordings, dwellings lacking hygiene (*n* = 19), needing repair (*n* = 27), with mold/mildew/fungi (*n* = 34), or with pests or vermin (*n* = 18), i.e., rodents and insects, e.g., bedbugs and triatomines (Appendix B: Table A1). None of the papers provide evidence of causation, i.e., that overcrowding leads to any dwelling condition characteristic(s). Still, several papers addressed the issue, providing information summarized in the synthesis section. Categorization of the records yielded 94 in category B and only six in category A (Figure 2), i.e., where the authors, beside presenting clear information on overcrowding and dwelling condition characteristics, also calculate their statistic association.

### 3.3. Data Extraction from Eligible Records in Category A

In the six category A studies [21,49,59,69,82,85] overcrowding associated with water damage and the following specific dwelling condition characteristics: mold, peeling paint cockroach, microorganism distribution in dust, cockroach allergen in dust, dust-mite allergen in dust, lead in dust, and cadmium in dust (Table 1). In addition, three characteristics showed trends towards increases, i.e., rodents (rats and mice), rotting wood, and leaks under sinks. These characteristics could coincide, e.g., Bradman et al. [21] reported the association of rodent or cockroach infestation with peeling paint, water damage, mold (rodent only), and level of cleanliness. About half of the studied families used pesticides in their homes, but the potential association of pesticides with overcrowding and other characteristics was not reported.

### 3.4. Data Extraction from Eligible Records in Category B

Some of the 94 category B papers described dwelling condition characteristics not previously mentioned, e.g., fomites and surfaces contaminated with infectious agents, and pests serving as vectors [9,62] for pathogenic bacteria, viruses, and amoebas. Characteristics sometimes coincide or are combined; for example, Huet et al. [50] used the following survey question to define need of repair: *‘‘Does your home have a problem with mold or is in need of major repairs (for example: a new roof, plumbing repairs, structural repairs)?’’*. Characteristics are sometimes even combined into an index [54,94]. Thus, Keall et al. [54] devised a respiratory hazard index that includes mold on indoor walls, fungi/mold on joists or bearers, and major or minor leaks in roofs. Concerning disrepair and physical characteristics, Quandt et al. [81] give many examples of injury hazards, divided into five types, three of relevance for hazardous dwelling condition characteristics, i.e., structural defects, electrical hazards, and fire hazards. This study points out that pest control measures increase with the level of housing disrepair reported [81]. One record state that it is common practice to use anti-fungal paints to treat mold [39]; another reference states that the presence of pests also increases exposure to the pesticides used to control infestations [87].

### 3.5. Synthesis of Eligible Records

**Figure 3:**
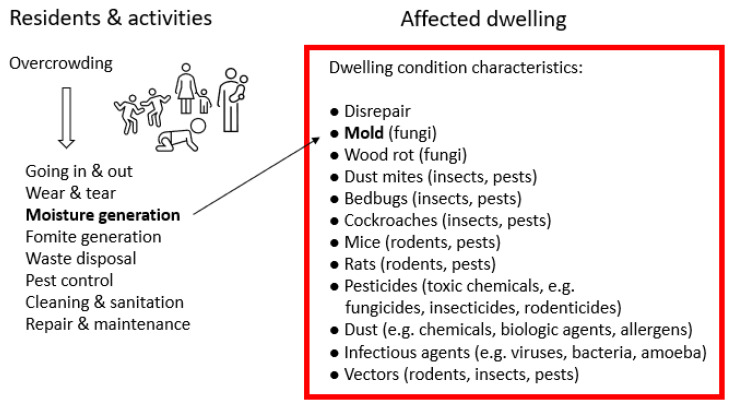
Extracted dwelling condition characteristics that may potentially be due to overcrowding and are of relevance for health. The bold text and thin arrow highlight one of the relations, in this case, that too many residents can lead to excess moisture generation and mold.

Overall, the papers define overcrowding in numerous ways and often focus on “socially deprived” residents. Overcrowded dwellings are often referred to as, e.g., “deteriorated”, “bad housing”, “inferior housing”, “substandard housing”, and “social housing”. These dwellings are in bad condition and can contain several hazardous dwelling condition characteristics. None of the records prove that overcrowding leads to these characteristics; however, many address circumstances pointing in that direction. For example, Howden-Chapman et al. report that mold associates not only with the number of residents but also with various activities, i.e., frequency of baths, showering and washing clothes [49]. Other activities influenced by overcrowding include going in and out, wear and tear, fomite generation, waste disposal, pest control, cleaning, sanitation, repair, and maintenance. We synthesize this information into a mind map (Figure 3).

As illustrated in Figure 3, overcrowding can lead to more or less of various activities. These changes may in turn affect the dwelling condition and lead to different characteristics. For mold to occur, moisture levels need to surpass a threshold for microbial growth. Other characteristics may not have thresholds, as when, for example, less efficient cleaning, waste disposal, maintenance, and pest control become beneficial for insects and rodents. Pests (and other environmental factors) may also relate to more persons going in and out, i.e., being introduced by the take-home route, particularly when people travel back from geographic regions where pests are more common [60]. Mold and pests lead to the use of various biocides [87], i.e., toxic chemicals. The resulting combinations of environmental dwelling condition characteristics may be very complex. Still, many characteristics can be amended at low cost [61], and solutions include education and changing activities and behaviors [38,49,75].

## 4. Discussion

Our initial hypothesis was that overcrowding in dwellings may lead to several biological, chemical, and physical dwelling condition characteristics of relevance for health. During extraction of such characteristics from reviewed literature, we found that many studies had quantitative data on both characteristic(s) and overcrowding but only a few presented data on their association. Thus, only a few studies were suited for analysis in line with systemic review. In order not to lose important information, we decided to map the relevant literature and to identify key observations in line with a scoping review. We encourage researchers to study and report the statistical associations, whenever possible, to increase the data base on overcrowding and dwelling condition characteristics of relevance for health.

Eight key observations evolved during the review work. First, while many individual papers describe one or a few hazardous characteristics, the records collectively provide evidence of many characteristics. Second, many characteristics may coincide in a dwelling (Table 1 and Figure 3). Third, biological characteristics such as mold and pests may often coincide with the use of toxic chemicals (biocides/pesticides). Fourth, overcrowding is described in numerous different ways, making it difficult to compare studies. Fifth, papers often focus on “socially deprived” groups. Sixth, while many papers present quantitative data on overcrowding and characteristic(s), very few present statistical information on their association. One reason for the lack of statistical analyses may be that the association is considered common knowledge, stemming from historical challenges that lead to the development of building codes and norms, including ventilation standards (not covered herein).

Thus, many OECD countries have solid experience with crowded city slums, representing various perceived threats to ordered society. Concerning health, some countries initiated national measures relating to housing already in the early 19th century, as the relation between housing conditions and health was recognized among public health practitioners in Europe and the United States [104]. On this background, the focus on “socially deprived” residents in many records and the numerous ways to describe overcrowding seem logical. Countries use different definitions of overcrowding and address the issue for different purposes, such as research, statistical reporting, regulation, and administration, e.g., for allocating housing and delivering social assistance [105].

Obviously, overcrowding does not necessarily lead to detrimental indoor environments, and dwellings may very well “deteriorate” without overcrowding, in many ways and for many reasons. Still, it is both logical and supported by the information in the reviewed records that overcrowding may lead to the characteristics we present. Two Swedish studies are telling in this context, reporting mold [82] and risk of mold [71], as far more residents than intended moved into carefully planned dwellings and city districts [71,82].

Importantly, from a public health perspective, mold and many other characteristics relate to activities that can be modified. Since human behaviors are outside the scope of this review, we did not cover habits that would influence characteristics or introduce new ones. For example, smoking and keeping pets, which would add pet allergens and carcinogenic tobacco smoke chemicals to dust, were not addressed.

Notably, our aim was not to assess the wide range of health effects attributed to overcrowding and/or dwelling condition characteristic(s), often mold. However, it is worth mentioning that overcrowding might also be beneficial [44]. One record reported that mold influences birth outcomes [32], but the most common health impact attributed to mold was respiratory symptoms and diseases, mainly asthma. Two studies on asthma dealt with psychologic stress [63,90].

This leads to the seventh key observation of our review, that it may be very difficult in epidemiological studies to understand what overcrowding represents and to disentangle all the potential environmental and social factors that could affect health outcomes, including behaviors. Several reviewed records emphasize complexity. In line with this, the 2018 WHO report on housing and health expresses concern regarding the causality of outcomes attributed to overcrowding, due to “the study designs, and the close association between social deprivation and crowding” [2]. Furthermore, a 2009 WHO report on dampness and mold present many potential environmental factors behind epidemiological associations of various health outcomes with water (dampness, humidity, moisture, and leaks) [106]. The presented biological and non-biological exposures partly overlap the characteristics described herein. However, neither of the two WHO reviews explicitly state that overcrowding, dampness, and mold may be interconnected [2,106]. Thus, our study not only complements the WHO reviews but also may bring new dimensions to the past and present epidemiological research, e.g., on asthma.

Our eighth and final key observation in relation to overcrowding is that pesticides/biocides are, reasonably, a major public health concern and have been for a long time. Several of the records we reviewed cover biocides used indoors against mold and vermin, e.g., [9,21,27,28,33,36,60,81,87,101]. The experts behind the WHO report on dampness and mold speculated that “the levels of semi-volatile compounds, such as pentachlorophenol (a wood preservative) and other pesticides, may also be elevated in damp indoor environments”. Two subsequent papers provided evidence for this, e.g., that chlorophenols were widely used in the 1950–1980s against mold indoors and fungi in damp constructions, giving rise to odor potent chloroanisoles that smell like mold [107,108].

Current public health advice is to limit application of pesticides, use pesticides least toxic to humans, and involve and educate residents in pest management [109]. Regarding pests, “deteriorated” dwellings, with or without mold, may contain several allergen sources, e.g., dust-mites, cockroaches, and rodents. This insight is important, e.g., for identifying allergies, handling respiratory symptoms of residents, and remediating indoor environments.

Concerning how this review was performed, our search strategy, based on “crowd”, may have missed relevant records that uses other terms, e.g., “residential density” or “household density”. Still, most of the relevant literature was likely captured as our complementing hand search for such terms yielded only a few additional studies. Further, we did not cover related terms, such “household size”, “family size” as these only represent one dimension of overcrowding, the other being spatial. We note that a 2012 WHO report on “Environmental health inequalities in Europe” points out that condensation or mold associate with poor social conditions associated with large family size and gives rise to humidity [110].

Societal actions to reduce overcrowding is likely to have a broad range of beneficial effects on wellbeing and health. Meanwhile, systematic reviews on studies dealing with health in relation to housing, overcrowding, and remediation often point to a lack of sufficient scientific rigor [2,111,112]. Most likely because the topic is very complex, involving many social and environmental aspects, making it inherently difficult to study. Even though we limited our review to specific environmental exposures in overcrowded dwellings, the results point to highly complex exposure situations.

The exposure complexity and connected health risks seem to have a global distribution. However, from a broader health perspective, it is worth pointing out that in countries where crowded housing is socially accepted, crowding might not necessarily lead to health issues in the same way as in countries with more narrow norms.

Finally, a systematic search today would most likely capture many studies on COVID-19; however, the topic of infectious diseases spread by fomites is already covered in our study.

## 5. Conclusions

To our knowledge, this is the first review based on the hypothesis that overcrowding in dwellings may lead to several dwelling condition characteristics of relevance for health. We present evidence supporting our hypothesis, suggesting that overcrowding in dwellings, defined in different ways, may associate with a broad range of hazardous characteristics, for example increased occurrence of mold, vermin, and allergens, as well as increased use of biocides (Figure 3). Each exposure may be recognized in public health, but our data highlight complex exposure situations.

Actions to prevent or reduce the health impact of the various exposures are important and should preferably include involving residents, for example by providing information on how they themselves might contribute to a healthier indoor environment.

While the occurrence of mold is often reported, it is important to note that it may coincide with other characteristics. Furthermore, both overcrowding and mold may covary with (indicate or be a proxy for) various social and behavioral circumstances.

Consequently, the epidemiological attribution of health effects to any exposure is difficult, with the attribution of causal relationships even more so. The complexity should be considered by scientists and practitioners dealing with overcrowding in dwellings.

A universal definition of overcrowding might be difficult to attain, considering differences in cultural norms, traditions, legislations, etc. Nevertheless, more harmonized terms and definitions would benefit future research in the area. Further, with respect to future research:

- More in-depth studies on the effects of overcrowding on the dwelling are needed. Such studies should preferably contain quantitative data, including definitions and measures of overcrowding, measures of effects, and relations between these parameters.
- Longitudinal cohort studies with quantitative data of the above type are needed. Such studies would facilitate the establishment of causal relationships between overcrowding and dwelling characteristics.
- Intervention studies are needed to investigate the impact of, for example, discussions and education of residents on how the indoor environment can be improved, changes in indoor climate control (ventilation, heating, air conditioning, etc.), and introduction of maintenance and repair plans.

## Figures and Tables

**Table 1:** Condensed description of the six studies in category A and extracted specific hazardous dwelling condition characteristics from each study.

Characteristics	Condensed Description
Mold Cockroach infestation Peeling paint Rodent infestation (NS) Rotting wood (NS) Leak under sink (NS) Pesticides (NR) Note: several characteristics associate with each other, e.g., mold and cockroach.	Bradman et al. (2004) [21]. Environmental assessments were carried out in 644 homes of pregnant Latina women and their children in an agricultural community in California between October 1999–2000. Participants were recruited via a health clinic serving predominantly low-income Latina clients. Most homes (85%) had four or more household members, and 69% of the homes housed at least one agricultural worker. A large portion (39%) of the homes were crowded, i.e., had more than 1.5 persons per room, and were in bad condition: 58% had peeling paint, 43% had mold, 25% had water damage, and 11% had rotting wood. About half of the families used pesticides in their homes, mainly pyrethroid insecticide sprays and powders. Cockroaches and rodents were present in 60% and 32% of the homes, respectively. Crowded living (more than 1.5 persons per room) was significantly associated with cockroach infestation (odds ratio (OR) 2.7, *p* < 0.01), peeling paint (OR 2.2, *p* < 0.01) water damage (OR 2.5, *p* < 0.05, and mold (OR 1.9, *p* < 0.05) and weakly (non-significantly) related to rodent infestation (OR 1.1) rotting wood (OR 1.2), and leak under sink (OR 1.1). There were also significant associations between rodent or cockroach infestation and a number of home conditions: peeling paint, water damage, mold (rodent only), and level of cleanliness.
Cockroach allergen Dust mite allergen	Leaderer et al. (2002) [59]. Dust samples were collected in the living areas of 999 homes of asthmatic children in southern New England, USA, 1996–1998, and analyzed for dust mite, cockroach, cat, and dog allergens. Data on maternal education, income, race, dwelling type (single or multi-family household), population density (people per square mile), and household density (people per room) were collected by home interviews and census data. Logistic regression analyses revealed significantly increased odds ratios for elevated levels of cockroach allergens but lowered levels of mite allergens in low income, black and Hispanic, multi-family, and crowded households (more than one person per room).
Mold Note: cockroach appears to be a common characteristic but is not measured.	Richter et al. (2018) [82] conducted a cross-sectional study among immigrant families in Sweden to assess the contribution of bad housing conditions to poor health. Families were recruited via identification of children with respiratory problems. In all, 130 families (650 individuals) were included. Data on living and dwelling conditions were obtained by the combination of questionnaires and home inspections. Eighty-four of the households were classified as crowded (more than one person per bedroom), and crowdedness was significantly associated with subjective as well as objective reports of dampness and mold. In addition, 416 of the 650 participants underwent skin prick tests against common aeroallergens, including molds, house dust mites, plants, and animal dander, and to cockroach antigen. Crowdedness did not significantly influence the children’s risk for being sensitized overall. However, participants showing sensitization against cockroach allergen (11 in total) were much more likely to live in an apartment with cockroach exposure (*p* = 0.006) than non-sensitized participants, and all homes with children sensitized against cockroach antigen (5 in total) had cockroach infestation in the past.
Mold	Howden-Chapman et al. (2005) [49] undertook a random telephone survey regarding mold and its risk factors in New Zealand houses. In total, 613 household responded (response rate 50.5%). Multivariate analysis revealed that a number of house construction and climate and behavioral factors were significantly related to reported mold, including older house, lack of sun exposure, no insulation, high locality rainfall, living in the north of the country and frequent baths, showers, and clothes washing. With respect to crowdedness, the analysis revealed significant positive relations between the prevalence of mold and number of residents, number of residents below 18 years old, and number of residents per bedroom. With respect to the latter, the prevalence of mold was 29% for less than 1 resident, 34% for 1–1.5 residents, and 48% for more than 1.5 residents per bedroom.
Microorganism distribution (fungi, dust mite, and bacteria) Note: pesticide use was measured, considered necessary to explain microorganism distribution, but results were not presented.	Rocchi et al. (2015) [85] studied the microorganism composition in dust collected from 3193 French dwellings 2011. Dwellings were recruited via a subsample (EBRA, microbiological environment, and allergic risk) of the Elfe cohort (a large cohort devoted to monitoring children’s development from birth to adulthood). The analyses included six fungal species, three families/genera of bacteria, and house dust mite. Data on 13 dwelling characteristics were collected, namely dwelling type (apartment or house), family situation (owner, tenant, and free), pets, dwelling renovation, pesticide use and potted plants (yes and no), daily housework time, floor covering in bathroom, kitchen and living room (tile, linoleum, parquet, and other), window number, floor number, and occupation ratio (inhabitants per m^2^). Of these characteristics, occupation ratio, dwelling type (house/apartment), and presence/absence of pets were significantly related to the microorganism distribution in the dwellings, although they only explained a small fraction of the variance (3%). Dust mite; Enterobacteriaceae; and to a lesser extent, *C. sphaerospermum* were mainly associated with occupation ratio.
Lead in house dust Cadmium in house dust	Meyer et al. (1999) [69] measured lead and cadmium in house dust collected for one year (starting October 1993–August 1994) from 415 dwellings located in different industrial areas in eastern Germany. Lead and cadmium deposition rates (expressed as µg/m^2^ per day) were significantly associated with area of residence, urban environment (park, side road, main road, and industrial), type of heating (central/district heating, gas, coal), year of construction, and crowding. Regarding crowding, the average deposition rates were 61% (lead) and 80% (cadmium) higher in sampling rooms with three or more persons, compared to rooms with no persons.

NS, not significant, NR, not reported.

## Data Availability

Not applicable.

## Appendix A. Search Strategy

PubMed and Scopus were searched (title, abstract, keywords, and mesh-terms), using the following search string:

*crowd* AND (indoor OR building OR household* OR home* OR dwelling OR residen* OR housing OR house*) AND (“united states” OR “united kingdom” OR germany OR australia OR canada OR italy OR japan OR france OR netherlands OR spain OR switzerland OR sweden OR “new Zealand” OR denmark OR poland OR ireland OR turkey OR israel OR portugal OR finland OR austria OR greece OR mexico OR belgium OR norway OR “czech republic” OR chile OR hungary OR estonia OR luxembourg OR lithuania OR iceland OR latvia OR “slovak republic” OR slovenia OR korea OR europe OR America OR colombia).

## Appendix B

**Table A1:** Overview of content in the 100 records included in this scoping review. Country codes are given according to ISO Alpha 3 standard. Country code refers to the location of the dwellings under study. Year refers to the publication year.

Main Dwelling Condition Characteristic(s) in the Abstract	Population(s) in Focus ^†^	Country Code ^‡^	Year	Reference
Allergens, respiratory irritants, infectious agents	n.d.	AUS	2001	[19]
Altered microbial exposure	n.d.	AUS	2019	[30]
Bedbugs	n.d.	USA	2009	[60]
Biological factors	Indigenous	Several	2017	[17]
Cleaning of dwellings	Immigrant Latino farmworker families	USA	2006	[28]
Cleanliness, pests	n.d.	USA	2013	[101]
Cockroach	n.d.	USA	2018	[102]
Cockroach, dust mite	n.d.	USA	2001	[93]
Contaminated surfaces, Hepatitis B virus	Eskimo	USA	1976	[78]
Contamination of surfaces, *S aureus*	n.d.	USA	2020	[79]
Deteriorated (no abstract)	Black families	USA	2010	[84]
Deterioration (i.e., peeling paint, holes in floor, broken windows)	Fragile families	USA	2010	[90]
Disrepair, allergens, toxicants	Indigenous, non-indigenous	AUS	2010	[27]
Disrepair, allergens, toxicants	Indigenous, non-indigenous	Several	2010	[26]
Disrepair, cockroaches, mold, rotting wood, rodents, water damage, peeling paint, pesticides, less clean	Immigrants, pregnant Latina women, low income	USA	2005	[21]
Domestic hygiene, non-functioning infrastructure	Aboriginal children	AUS	2009	[67]
Dust	n.d.	GBR	1994	[92]
Dust from hobbies	n.d.	CAN	1989	[47]
Dust mite allergens, cockroach allergens	Asthmatic children	USA	2002	[59]
Dust mite, fungi, molds, bacteria	n.d.	FRA	2015	[85]
Dust, cockroach allergen	n.d.	USA	1996	[88]
Dust, house dust mite, allergens	n.d.	GBR	2007	[44]
Endotoxin	n.d.	Several	2012	[24]
Environmental hygiene	Roma, non-Roma, unemployed	HUN	2014	[46]
Filth (no abstract)	Inhabitants in slum conditions	CAN	2004	[18]
Hazards	n.d.	GBR	1998	[31]
House dust mites, fungi, maintenance	n.d.	Several	1996	[48]
House dust mites, insufficient cleaning	n.d.	TUR	2007	[11]
Hygiene	Indigenous	AUS	2019	[42]
Hygiene	Indigenous	AUS	2010	[68]
Hygiene	Aboriginal children	AUS	2009	[41]
Hygiene	Latina immigrant women	USA	2004	[38]
Hygiene, cleanliness	n.d.	GBR	2008	[45]
Hygiene, contaminated fomites, *C diphteriae*	Inhabitants in skid road conditions, mainly alcoholics	USA	1989	[43]
Hygiene, *Helicobacter pylori*	n.d.	POL	2014	[98]
Hygiene, infrastructure function	Indigenous	AUS	2010	[13]
Hygiene, sanitation	n.d.	DEN, SWE	2007	[91]
Hygieneic state of houses	Indigenous	AUS	2012	[14]
Insanitary	Inhabitants in areas of extreme poverty	IRL	1992	[4]
Lack of hygiene (attendant bacteria and fungi)	Inhabitants in areas of extreme poverty	CHL	2001	[5]
Leaking roof	n.d.	Several	2015	[20]
Major repairs needed	Inuits	CAN	2020	[83]
Moisture damage	n.d.	SWE	2019	[71]
Mold	First nations	CAN	2012	[75]
Mold	n.d.	DEU	2010	[23]
Mold	n.d.	DEU	2017	[100]
Mold	n.d.	FIN	2017	[97]
Mold	n.d.	FRA	2020	[37]
Mold	Italians, foreigners	ITA	2012	[22]
Mold	n.d.	NZL	2011	[40]
Mold	n.d.	NZL	2005	[49]
Mold	Maori, non-Maori	NZL	2019	[51]
Mold	Unemployed	NZL	2012	[54]
Mold	Maori, Pacific	NZL	2013	[55]
Mold	n.d.	NZL	2018	[65]
Mold	Maori, Pacific	NZL	2017	[74]
Mold	n.d.	Several	2007	[10]
Mold	n.d.	SWE	1991	[73]
Mold	Immigrant families	SWE	2018	[82]
Mold	n.d.	USA	2018	[32]
Mold or mildew	n.d.	NZL	2016	[96]
Mold, allergens, pathogenic organisms	n.d.	GBR	2015	[99]
Mold, allergens, pollutants	n.d.	USA	2006	[63]
Mold, mildew, allergens, pesticides, structural deficiencies	Farmworkers	USA	2015	[81]
Mold, need of major repairs	Inuits	CAN	2011	[70]
Mold, pests, plumbing leaks	Mexican immigrant families	USA	2009	[61]
Mold, poor repair	First nations	CAN	2011	[58]
Mold, structural deficiencies	First nation	CAN	2012	[64]
Mold, water leakage, cleaning	Low-income people	KOR	2014	[89]
Mold/moisture	n.d.	Several	2006	[35]
Molds, fungi	n.d.	FRA	2008	[86]
Moldy housing conditions	Socially deprived residents	GBR	1999	[16]
Need major repairs	Inuit children	CAN	2015	[56]
Need of major repairs	Inuit children	CAN	2010	[29]
Need of major repairs	Inuits	CAN	2012	[50]
Need of renovation	n.d.	DEU	2004	[80]
Pollutants	n.d.	Several	2013	[33]
Pollutants, microbial products, allergens	Ethnic minorities, poor residents	USA	2010	[52]
Pollution	Residents in underserved community	CHL	2014	[66]
Poor dwelling conditions	Aboriginals	AUS	2016	[7]
Poor housing conditions	Aboriginal children	AUS	2005	[15]
Poor housing conditions, hazards	Migrant farmworkers	USA	2015	[95]
Poor hygiene, Helicobacter pylori, housefly	n.d.	Several	2000	[62]
Rats, rat fleas, vectors, *Rickettsia typhi*, insecticides, rodenticides	n.d.	USA	2021	[9]
Repair	First nations, Inuit children	Several	2012	[57]
Repair	Fragile families	USA	2011	[94]
Repair, Infestations	n.d.	CAN	2015	[87]
Repair, moisture damage	Social housing residents	CHL	2019	[39]
Repairs	Indigenous	AUS	2021	[34]
Rot in window frames, a leaky roof	Housing deprivation	ESP	2008	[72]
SARS-CoV-2 on surfaces of fomites	n.d.	Several	2020	[12]
Sedimented dust, lead, cadmium	n.d.	DEU	1999	[69]
Sewage, pests, vermin, mold, mildew	Indigenous	AUS	2018	[6]
Structural hazards	n.d.	USA	2019	[53]
Structural problems, mildew, vermin	Aboriginals	AUS	2018	[8]
Structural problems, roof leaks	Latino farmworker families	USA	2007	[36]
Synanthropic rodents, sanitary conditions, *Leptospira* spp.	n.d.	COL	2013	[103]
Unhygienic conditions	Italians, foreigners	ITA	2020	[25]
Unsanitary conditions, toxins, pollutants	n.d.	USA	2003	[77]
Unsanitary residential conditions	Low-income residents	COL	2017	[76]

^†^ n.d., not determined. No obvious focus on “socially deprived” residents or ethnic dimensions. ^‡^ Several, locations in several countries of the dwellings under study, e.g., in multi-center papers and reviews.

**Table A2:** Expressions used to describe or define crowdedness in the 100 records included in this scoping review. Country codes are given according to ISO Alpha 3 standard. Country code refers to the location of the dwellings under study. Year refers to when the dwellings were investigated.

Categories (in Italics) and Expressions	Country Code	Year	Reference
*Persons per household*			
Number of house residents: 1–2, 2–4, 5 or more.	NZL	2000	[49]
Mean number of residents in the homes.	CHL	2014 ^†^	[66]
Average number of people per household.	AUS	2004–2005	[14]
Number of other siblings in the family.	GBR	1991–1992	[16]
Domestic crowding: number of people in the household not controlling for the size of the home. C1: 5 or more, C2: 1–4.	USA	1989 ^†^	[47]
*Persons per dwelling area*			
Number of individuals residing in the dwelling in relation to its surface area higher than the value envisaged by articles 2–3 of the 1975 Ministerial Decree (<14 m^2^ per person för de first four, <10 m^2^ per person for each of the following).	ITA	2012–2016	[25]
Number of persons per m^2^. Crowding: those above the 75th percentile.	RUS	1988–1999	[35]
Crowded housing conditions: <21 m^2^ per capita.	DEU	2003–2006	[23]
Floor area per person: <10 m^2^, more than 20 m^2^.	POL	2002–2003	[98]
The home has at least 100 ft^2^ of living space per person.	USA	2013–2016	[53]
<20 m^2^ per person.	DEU	2014–2015	[100]
Occupation ratio, i.e., occupants per m^2^.	FRA	2011	[85]
Total area of house divided by number of persons who permanently resided therein.	CAN	2007–2008	[58]
Crowding factor calculated as house size divided by number of family members (27.9 m^2^ per person on average for households in the sample).	KOR	2010–2011	[89]
*Persons per room*			
People per room. The US Census considers >1 person per room to be crowded.	n.a. ^‡^	n.a. ^‡^	[33]
Less or more than 1 person per room. For household crowding, Statistics Canada’s definition of more than one person per room was used, where rooms included bedrooms, kitchen and living room.	CAN	2007–2008	[29]
Number of persons per room.	GBR USA, CAN USA NZL CAN DEU	1937–1939 1988–1999 1996–1998 2002–2004 2014–2015 2014–2015	[45] [35] [59] [40] [83] [100]
More than 1.5 persons per room.	GBR USA	1988 1999–2000	[92] [21]
Crowded household: more than 1 person per room.	USA	2001	[28]
Crowding: average occupancy of 1.01 or more, extremely crowded: 1.51 or more persons per room.	USA	2015 ^†^	[81]
Not crowded: <0.5, crowded: 0.51–1.5, severely crowded: ≥1.51 persons per room.	USA	1988–1994	[93]
Number of persons per room.	GBR	1991–1992	[16]
More than 1 person per room.	GBR GBR USA	1991 1992 2004	[32] [31] [36]
Canada’s household crowding definition of more than 1 person/room was used where rooms included bedrooms, kitchen, and living room.	CAN CAN	2007–2008 2007–2008	[50] [70]
Persons per room, more than 1, more than 1.5.	CAN	2006	[56]
Number of persons living in a dwelling divided by number of rooms in the dwelling, excluding bathrooms or storage areas.	CAN	2007–2008	[58]
Overcrowding: >1 person per room, severe overcrowding: >1.5 persons per room.	USA	2005–2007	[61]
Overcrowding: occupancy level exceeding more than one person per room.	CAN	n.a. ^§^	[75]
Number of persons in household divided by number of rooms. Households then dichotomized into low crowding (two thirds of the households) and high crowding.	DEU	1999	[80]
Number of household members divided by number of household rooms.	DEN, SWE	1999–2002	[91]
*Persons per bedroom*			
Number of residents per bedroom; <2 (reference), 2–4, >4.	AUS	2005 ^†^	[15]
Child shares bedroom.	CHE	1988–1999	[35]
Number of persons per bedroom. Crowding: those above the 75th percentile.	USA, CAN	1988–1999	[35]
Number of residents per bedroom.	NZL	2000	[49]
The mean number of persons per bedroom.	AUS	2002	[67]
People per bedroom.	USA	2012–2015	[79]
Household members per bedroom.	NZL	2007–2008	[54]
Overcrowding exists, with community members reporting 3-bedroom homes sheltering 9–10 people.	CAN	2012 ^†^	[64]
On the basis of the housing occupancy standard of a maximum of two persons for each available bedroom (Australian Bureau of Statistics, 2000),	AUS	2010 ^†^	[68]
84 households (65%) were classified as crowded: more than one person per bedroom.	SWE	2009–2018	[82]
*Persons at certain age*			
Number of residents below 18 years.	NZL	2000	[49]
Number of rooms less than the number of adults >16 years.	ESP	1998	[72]
Number of small children (<5 years) at home: 0, 1, 2, >2.	SWE	1988–1989	[73]
*Persons sharing bed*			
Functional overcrowding because children share room or bed because they need to keep warm.	NZL	2012	[55]
*Crowding at area level*			
Average dwelling crowding for a local area ex-pressed as: $CI=\sum \left(\frac{P}{B}\times \frac{F}{T}\right)$ (P = no. of persons usually resident, B = no. of bedrooms, F = frequency of no. of persons by no. of bedrooms combination, T = total no. of combinations).	AUS	1996	[19] ^¶^

^†^ Year of publication, year of investigation not stated. ^‡^ n.a., not applicable, review covering several countries and years. ^§^ n.a., not applicable, review covering several years. ^¶^ The appendix to the paper has a compilation of definitions of crowding.
